# Development of Semi-Automatic Dental Image Segmentation Workflows with Root Canal Recognition for Faster Ground Tooth Acquisition

**DOI:** 10.3390/jimaging11100340

**Published:** 2025-10-01

**Authors:** Yousef Abo El Ela, Mohamed Badran

**Affiliations:** Department of Mechanical Engineering, School of Sciences and Engineering, The American University in Cairo, New Cairo 11835, Egypt; yousef.ela@aucegypt.edu

**Keywords:** image segmentation, region growing, watershed, ground truth, CBCT, endodontics, GAN, nnU-Net

## Abstract

This paper investigates the application of image segmentation techniques in endodontics, focusing on improving diagnostic accuracy and achieving faster segmentation by delineating specific dental regions such as teeth and root canals. Deep learning architectures, notably 3D U-Net and GANs, have advanced the image segmentation process for dental structures, supporting more precise dental procedures. However, challenges like the demand for extensive labeled datasets and ensuring model generalizability remain. Two semi-automatic segmentation workflows, Grow From Seeds (GFS) and Watershed (WS), were developed to provide quicker acquisition of ground truth training data for deep learning models using 3D Slicer software version 5.8.1. These workflows were evaluated against a manual segmentation benchmark and a recent dental segmentation automated tool on three separate datasets. The evaluations were performed by the overall shapes of a maxillary central incisor and a maxillary second molar and by the region of the root canal of both teeth. Results from Kruskal–Wallis and Nemenyi tests indicated that the semi-automated workflows, more often than not, were not statistically different from the manual benchmark based on dice coefficient similarity, while the automated method consistently provided significantly different 3D models from their manual counterparts. The study also explores the benefits of labor reduction and time savings achieved by the semi-automated methods.

## 1. Introduction

Alongside technological advancements in medical radiography, another complementary tool has also been undergoing significant development, namely image segmentation. Several techniques have been developed over the years to deal with Digital Imaging and Communications in Medicine (DICOM) which has been the common standard for years [1]. DICOM has made significant contributions to multiple medical fields, including dentistry as one of the adopters of this standard [2]. Image segmentation plays a crucial role in the medical industry, as it enables accurate diagnosis and treatment planning by dividing an image into distinct regions of interest. This technique is beneficial in various ways, including enhancing diagnostic accuracy, streamlining treatment planning, and improving patient outcomes.

One significant application of image segmentation in dentistry is in VR-based simulators. These simulators use image segmentation to create realistic and detailed models of teeth and dental structures, allowing dentists to practice and refine their skills in a highly immersive and interactive environment [3]. In endodontics, image segmentation is essential for visualizing and understanding the internal anatomy of teeth, which is critical for root canal therapy. For instance, a study investigated root canal detection using augmented reality, and it was found to have a generally greater sensitivity when detecting root canals in molars and premolars [4].

### 1.1. Current State of Segmentation and Problems

Various automated techniques have been proposed over the last few years to facilitate image segmentation in medicine and dentistry imaging. For two-dimensional segmentations, and specifically for dentistry, Wu et al. [5] present a novel method for evaluating orthodontic parameters using dental panoramic radiographs that is structured into the following four key stages: image preprocessing, model training, tooth segmentation, and assessment of orthodontic parameters. Initially, images are normalized and enhanced, followed by using training models to accurately identify tooth shapes and contours. The final stage involves automatic assessment of orthodontic parameters, yielding results that closely align with evaluations made by orthodontists. Experimental findings demonstrate that the method achieves a high accuracy, with metrics such as the Dice similarity coefficient ranging from 0.87 to 0.90, indicating its potential to enhance objective treatment evaluations in orthodontics. Another utilization of panoramic X-rays is performed by Lai et al. [6], where they introduce a top-down deep learning architecture for human identification that integrates an improved channel attention module and a learnable connected module, enabling it to effectively extract and emphasize interdependent features across various layers. This approach allows for more accurate matching of dental images, achieving a rank-1 identification accuracy of 87.21% and a rank-5 accuracy of 95.34% on a dataset of 1168 images from 503 subjects. The extensive experimental results showcase the architecture’s potential utility in forensic odontology for reliable human identification. Although these methods, among others, have achieved good results, their input modalities are two-dimensional. Once the requirement becomes obtaining 3D volumes, such workflows need to be more robust to output acceptable results, and, therefore, they require more training data.

Many other segmentation techniques have been proposed to achieve 3D segmentation [7,8,9,10,11,12,13,14,15]. However, the focus of these studies is on the segmentation of the root canal, since endodontics is not the main target of the results. Therefore, studies have segmented the tooth as a solid object with no cavity where the pulp chamber or root canals should be. For endodontics-oriented approaches, Dumont et al. [16] propose machine learning approaches utilizing U-Net [17] and ResNet to automatically segment and classify dental root canals and crowns from Cone Beam Computed Tomography (CBCT) scans and digital dental models, respectively. The shortcomings of this work include the low sensitivity of root canal segmentation due to limited training data, CBCT field of view, class imbalance, as well as the need for further learning to handle digital dental models with missing teeth in crown segmentation. Deleat-Besson et al. [18,19] employ deep learning techniques, including a U-Net architecture for root canal segmentation and labeling, achieving an F1-score of 0.84, and a multi-view approach for 3D shape analysis in dental model segmentation with an accuracy of 0.9. This work acknowledges the need for further research to enhance the robustness and accuracy of segmentation models, especially in handling variations and complexities in dental anatomy and improving model generalizability, performance, and clinical applicability, which could benefit from more training data.

Wang et al. [20] utilize multi-task learning for tooth and pulp segmentation, achieving effective tooth instance segmentation and pulp region segmentation. Issues with this work include difficulty in segmenting the pulp region due to varied shapes, low-contrast boundaries, and metal artifacts in CBCT images, especially with a limited number of training samples. Zhang et al. [21] propose an automated and accurate method for root canal segmentation from dental CBCT images using a 3D U-Net [22] architecture with a global image encoder for localization and a local region decoder for accurate segmentation. Results could be improved with a larger dataset, as the study is conducted on only 78 images. Duan et al. [23] present a two-phase deep learning solution for accurate tooth and pulp cavity segmentation from CBCT images, highlighting the challenge of limited labeled data for training, which can impact the efficiency of the network.

An important addition to the original U-Net architecture is the work by Isensee et al. [24]. This paper differs in its automated, adaptive approach to pipeline configuration, rather than relying on manually defined architectures and hyperparameters. While the original U-Net proposes a specific network architecture, nnU-Net analyzes the training dataset and automatically determines optimal preprocessing steps, architecture modifications, and training settings for each application, allowing it to generalize better and streamline deployment over a wider variance of datasets, whereas standard U-Net requires user expertise and tailoring for the best results on new datasets. Dot et al. [25] implement nnU-Net by adapting it for the automatic segmentation of dento-maxillo-facial CBCT and CT scans, creating the DentalSegmentator model. The authors leverage the self-configuring pipeline of nnU-Net to robustly segment multiple anatomical structures, including the upper skull, mandible, teeth, and mandibular canal, without the need for manual parameter tuning for their dental imaging data. Their approach involves training nnU-Net on a large, diverse dataset of dental scans and integrating both the model and processing pipeline into open-source tools, enabling easy reproducibility.

These advancements in automated image segmentation have the potential to significantly improve the precision and effectiveness of dental treatments. However, one common issue facing these deep learning methods is the lack of ground-truth-labeled data for models to train on, which is mainly due to the fact that manual segmentation is a time-consuming, tedious, and subjective process [26].

**Figure 1:**
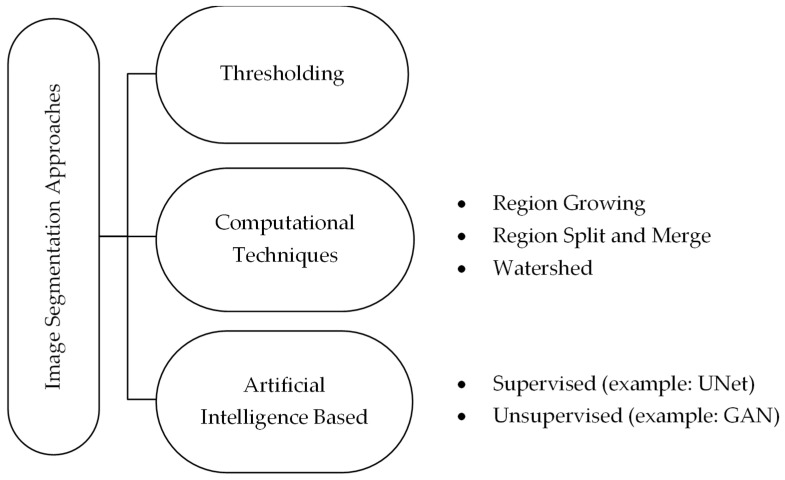
Classification and examples of image segmentation techniques used in dentistry.

Image segmentation can be performed using various techniques, including thresholding, region growing, and AI-based methods like convolutional neural networks. Thresholding involves setting a pixel brightness level, which can be automatically chosen, as the cutoff for selection, resulting in binarization of the image [27,28]. Region-based methods like region growing [29] and WS algorithms [30] leverage seed points, gradient information, and topographic map treatment to effectively delineate regions even in the presence of intensity changes, complex structures, and irregular shape boundaries [29,30]. AI-based techniques, particularly convolutional neural networks, have become popular for medical image segmentation due to their robustness in handling artifacts and ability to recognize patterns [31,32]. Level set, graph cut, atlas-based, and deep Boltzmann machine are all among the techniques under this last category [31,33]. Figure 1 mentions a few of the techniques used in image segmentation in the medical sector in general and the dental field in particular.

Automating tooth segmentation faces challenges such as complex dental structures, low-quality imaging, limited training datasets, and the obscuring of tooth boundaries by surrounding tissues. Issues like the proximity of adjacent teeth, variations in arrangement, and CBCT image problems, such as low resolution, noise, and artifacts, further complicate the process. Researchers address these challenges through preprocessing to improve image quality and by developing deep learning models, which require large, accurate labeled datasets to perform effectively. Recent advancements in data augmentation techniques, such as those discussed in Section 1.2.2, have helped enhance training datasets, improving segmentation outcomes [34].

Moreover, the generalizability of the already trained models mentioned earlier is an issue, since deep learning models trained on one dataset may not perform well on other datasets, even if they are from the same anatomical site or vendor’s scanners [35]. Therefore, the need for continuous availability of ground truth data is greatly beneficial, as commonly stated in the previous work, to enhance performance. Ground truth data plays a pivotal role in the development and validation of deep learning models for teeth segmentation from CBCT images. It serves as the reference standard against which the performance of automated segmentation algorithms is evaluated. By providing accurate annotations of tooth structures, root canals, and crown shapes, ground truth data enables the training of deep learning models to learn the intricate patterns and features necessary for precise segmentation. The quality and reliability of ground truth data directly impact the effectiveness and generalizability of segmentation models, ensuring that they can accurately identify and delineate dental anatomy in diverse clinical scenarios. Therefore, the availability of high-quality ground truth data is essential for enhancing the accuracy, robustness, and clinical applicability of deep learning models in teeth segmentation from CBCT images. Manual segmentation by experts is currently being used as the ground truth gold standard on which deep learning models are trained [11]. Although the results are satisfactory in terms of accuracy, they depend heavily on the clinician’s experience with tooth anatomy and familiarity with the software used. Even with experienced clinicians and fast workflows, the process of manually segmenting teeth with great accuracy to provide more training samples is greatly time-consuming and labor-intensive [23].

### 1.2. Related Work

In pursuing the objective of this dataset insufficiency challenge in deep learning model training, two approaches are currently being implemented, namely semi-automated segmentation and Generative Adversarial Networks (GANs).

#### 1.2.1. Semi-Automated Workflows

**Figure 2:**
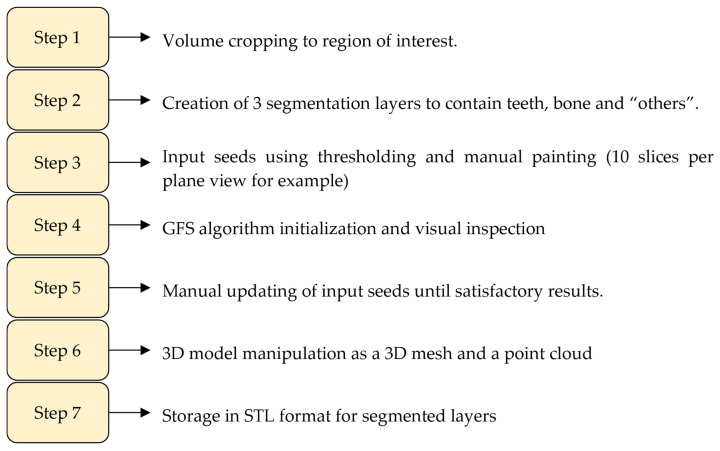
Summary of the steps and work carried out by Verykokou et al. in dental image segmentation for periodontal application [36].

In an attempt to achieve the goal of faster yet accurate segmentation, Verykokou et al. [36] propose a methodology for segmentation that involves a semi-automatic approach combining thresholding, manual definition of samples, and region growing using the GFS method in 3D Slicer software version 4.11.20210226 [36,37,38]. The region of interest is first cropped from the CBCT dataset, and the following three segments are defined: teeth, alveolar bone, and “other”. Samples for these segments are manually defined in a subset of CBCT images using data-dependent thresholds as masks to guide the manual definition process. The GFS method is then initialized, and the results are visually inspected and corrected, if necessary, by iterating over the input seeds, repeating the process until satisfactory segmentation is achieved. The segmentation results for teeth and alveolar bone are converted to 3D models in STL format, which are further processed in Geomagic Wrap 2017 commercial software [39] for point cloud editing, mesh creation, and optional merging of the teeth and alveolar bone models into a single 3D model. This semi-automatic approach aims to leverage the speed of thresholding and region growing while maintaining the flexibility and accuracy of manual refinement, ultimately generating high-quality 3D models of teeth and alveolar bone from CBCT data for various dental applications. The concluded workflow of this work is summarized in Figure 2. Issues with this work include disregard of the root canal and treating the tooth as one solid object, which is satisfactory for the authors’ use case of designing 3D scaffolds for periodontal regeneration. Micro CT scan is also suggested to improve outcomes due to its higher accuracy than CBCT. The authors also encourage the verification of this method on other datasets.

**Figure 3:**
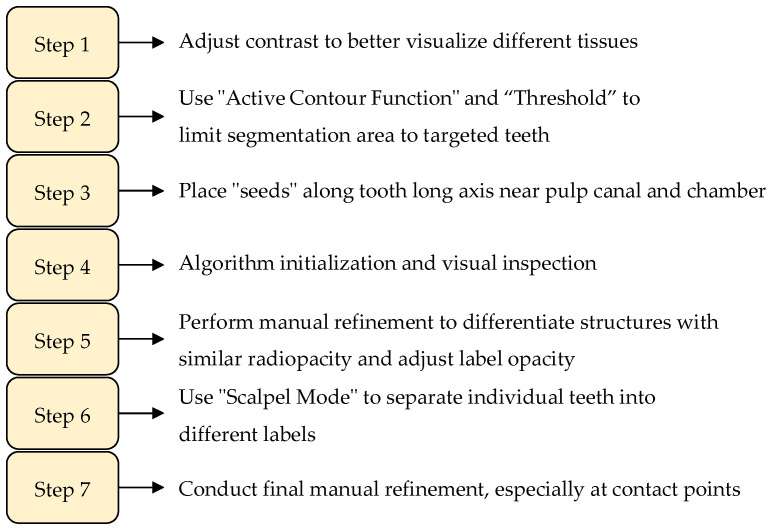
Summary of the steps and work carried out by Morell et al. in dental image segmentation with root canal recognition [40].

In another work on teeth segmentation, Morell et al. develop a workflow that considers the root canal as a separate segmentation layer [40]. The workflow begins by opening a CBCT DICOM file in ITK-SNAP software [41] and adjusting the contrast to better visualize the different tissues. The area of interest, such as the lower incisors and canines, is then zoomed into. The “Active Contour Function” or “Snake” is used to limit the segmentation area to the targeted teeth. The “Threshold” segmentation method is selected, and the upper threshold is set to the maximum while the lower threshold is adjusted individually for each patient, typically between 500 and 1500. “Seeds” or bubbles are then placed along the long axis of the teeth near the pulp canal and chamber to allow the segmentation to expand and automatically include the hard tooth tissues. After automatic segmentation, manual refinement is necessary to differentiate between structures with similar radiopacities, like bone and cementum. This is achieved by adjusting the label opacity and using the “Paintbrush Mode” to add or remove areas in the three anatomical planes. The “Scalpel Mode” is then utilized to separate individual teeth into different labels, followed by a final manual refinement, especially at the contact point areas. The software then calculates the volume of each segmented tooth label in mm^3^. The study includes a few CBCT imaging-specific problems, as well as the particular configurations used during CBCT collection. The field of view, voxel size, partial-volume effect, surrounding artifacts, scatter X-rays, subjectivity in the segmentation process, proximity of surrounding structures, and patient movement during imaging acquisition are a few of the reported CBCT issues that could have an impact on the segmentation results. Thus, there might be a lot of variation in the results’ correctness. An additional constraint is the duration of the procedure. For a busy practitioner, manual refinement may not be helpful because it is a rather time-consuming technique. Moreover, using the authors’ performance metrics, the accuracy ranges from “good to excellent”, which might not be well suited for accuracy-dependent use cases. A summary of the described workflow is shown in Figure 3.

Although the previous two works provide somewhat acceptable results according to their authors, their metrics of verification are not quite quantitative enough to assess the usability of these workflows as a replacement for complete manual segmentation.

#### 1.2.2. Generative Adversarial Networks

GANs play a crucial role in augmenting datasets that are often limited in size and diversity. Available datasets for training deep learning models frequently suffer from insufficiency and imbalanced class distributions. In response to these challenges, researchers have increasingly turned to GANs as a means of generating additional images, thereby enhancing the training process for deep learning models tasked with segmentation and classification, with the earliest example being Luc et al. in 2016, who employ GANs for semantic segmentation [42]. By generating synthetic images that reflect common and rare conditions, GANs can help create a more balanced dataset, allowing deep learning models to learn more effectively and generalize better across different scenarios. The original GAN framework introduced by Goodfellow et al. [43] contains the following two networks: a generator, which creates synthetic images, and a discriminator, which evaluates their authenticity by distinguishing real images from generated ones. This adversarial training process continues until the generator produces images that are indistinguishable from real data, effectively enriching the dataset with diverse samples.

Various GAN architectures have been employed to create a more specific or diverse framework depending on the targeted application. Mirza and Osindero [44] develop Conditional GANs, which generate images based on specific class labels and is quite popular when dealing with medical image augmentation [45]. Deep Convolutional GANs utilize convolutional layers for improved image quality [46]. CycleGANs is another improvement, which facilitates image-to-image translation without requiring paired training examples. These architectures, among others, have demonstrated significant success in generating high-quality synthetic medical images across various modalities such as MRI, CT scans, X-rays, and ultrasound [47].

For dental application of GANs, a few examples can be observed. Tian et al. [48] use a Wasserstein GAN to provide comprehensive dental inlay morphologies that are capable of restoring a tooth’s masticatory actions. This approach addresses challenges in generating anatomically accurate dental restorations, particularly for complex defects. Experimental results indicate that DAIS effectively handles extensive areas of missing tooth structure. In another work, Tian et al. [49] utilize a GAN to effectively generate accurate representations of gingival contours for patients with partial tooth loss. The proposed method focuses on improving the precision of the reconstructed margins by employing a dual-discriminator approach, which enhances the quality of the generated outputs by assessing both local and global features of the gingival line. Their results demonstrate that the framework achieves a high accuracy in reconstructing gingival margins. Another approach is known as DentaGAN, which combines the strengths of GANs with advanced segmentation techniques to enhance the delineation of teeth and surrounding structures in dental images. The model employs a unique attention mechanism to focus on important features, improving segmentation accuracy and robustness against varying tooth morphologies [50]. GANs have been widely implemented not only to augment datasets, but to denoise and enhance metal or motion artifacts and shades which can significantly affect a deep learning segmentation model [51,52,53,54,55,56].

Despite their advantages, the use of GANs for data augmentation is not without limitations. One significant challenge is mode collapse, where the generator produces a limited variety of outputs and fails to capture the full diversity of the target distribution. This can lead to models trained on such datasets performing poorly due to a lack of exposure to varied examples. Additionally, the training process can be unstable and sensitive to hyperparameter settings, making it difficult to achieve convergence between the generator and discriminator [45]. Regarding the ethical aspect, many studies assess the feasibility of GAN models without adequately addressing their effectiveness in real-world medical settings, where adherence to medical standards is essential, and, therefore, expert visual assessment of these GAN images is still needed [45,57]. This makes gaining acceptance from clinicians and patients regarding the quality of GAN-generated data pose a challenge, particularly given concerns about the potential generation of misleading or irrelevant disease information [57]. More importantly, in order to create annotated or classified GAN-generated datasets to act as ground truth for further deep learning models, the discriminator network still needs manually annotated ground truth data to be able to compare the generator’s classification outputs. Even after the generation of these images, studies such as those by Chi et al. and Shen et al. still use 50% and 46% manually annotated images in their training data, respectively [58,59].

These drawbacks do not necessarily dismiss the potential of GANs in the future of image augmentation and/or segmentation; however, they certainly highlight the current persistent need for manual image segmentation alongside the development of GANs and other innovative deep learning approaches.

In this paper, two semi-automatic workflows, using GFS and WS in 3D Slicer, are presented to significantly reduce the time spent manually segmenting teeth with root canal recognition, with minimal to no sacrifice in the accuracy of the output segmentation labels. Although there exist more sophisticated and automated workflows for this process, large, annotated ground truth datasets are essential for their training, especially when trying to generalize models to different datasets from the one they were trained on. The main objective of this work is to develop a streamlined framework that could be used to bolster the accessibility of available annotated datasets to be used as ground truth data for more deep learning automated approaches, with the benefit of major time savings, specifically targeted toward endodontics-oriented applications.

## 2. Materials and Methods

3D Slicer version 5.8.1 served as the software tool to develop a benchmark 3D model using complete manual segmentation, as well as other 3D models from other proposed workflows. 3D Slicer is not a dentistry-oriented segmentation software unlike some other programs such as BlueSky Plan [60], Relu^®^ Creator [61], Diagnocat [62], Cephx [63], and Exocad [64]. Despite some of these other tools offering automatic segmentation for teeth, 3D Slicer was chosen in this paper, as it offers a large variety of community-created plugins and tools, and, overall, it is a more versatile tool to develop a general workflow that can be applied to other types of CT scans. Moreover, most automatic tools do not offer manual refinement over already segmented models, which the user might not completely approve of. 3D Slicer is also more accessible than the other software mentioned, as they are only offered through a payment plan, while 3D Slicer is completely open-source. The tested methods were performed using a ConceptD 3 Ezel laptop (Acer, New Taipei City, Taiwan) running Windows 11 via an i7-10750H processor (Intel, California, United States), 16 GB of RAM, a GTX 1650 Ti (NVIDIA, California, United States), and an external mouse as the input device. This study was approved by the American University in Cairo’s Institutional Review Board (IRB). A waiver of consent for participation was also approved by the American University in Cairo’s IRB due to this study being carried out retrospectively and the preserved anonymity of the participant.

### 2.1. Datasets

Segmentation was performed on 3 separate CBCT DICOM images from an online database [65]. This was to ensure the independence of the method from the scanning parameters.

**Figure 4:**
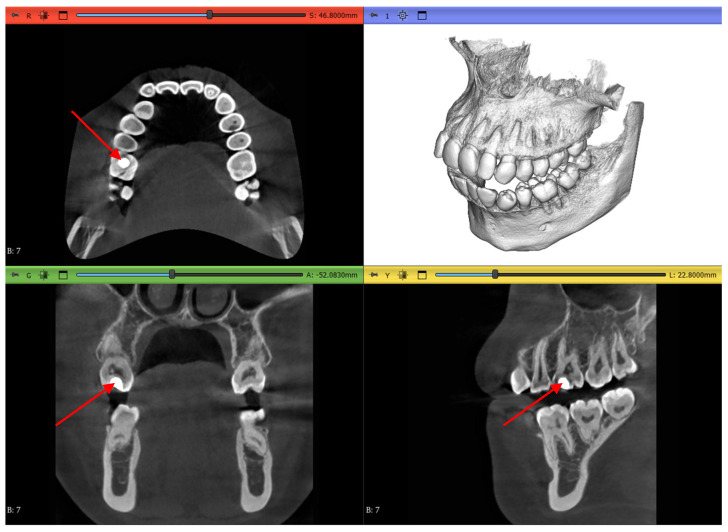
Dataset 1 of the study with red arrows showing the amalgam restoration.

Dataset 1 is the most straightforward of all, with a teeth spacer to avoid overlapping in the scans and no abnormalities except an amalgam restoration, as shown in Figure 4 by a red arrow, in a maxillary first molar which is not of interest to this study.

**Figure 5:**
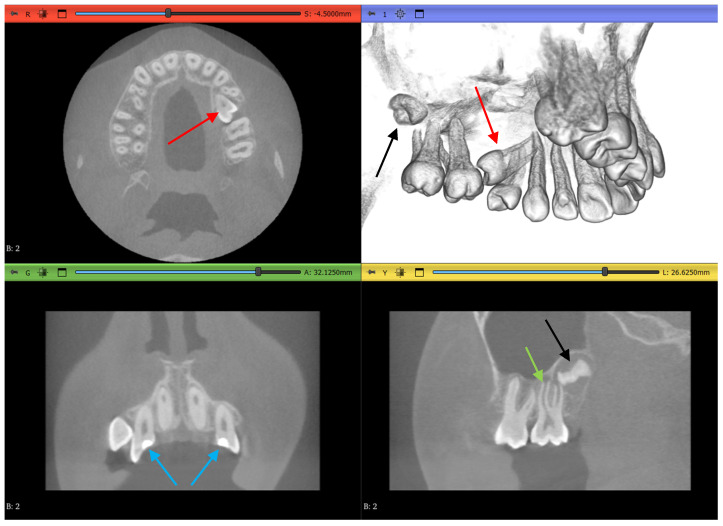
Dataset 2 of the study showing open apexes by a green arrow, restorations by blue arrows, impacted molar by red arrows, and unerupted molar by black arrows.

Dataset 2 is slightly more challenging, as it does not contain mandibular teeth. Figure 5 shows that the maxillary teeth are newly erupted with open apexes, as shown by a green arrow, have restorative operations shown by blue arrows, an impacted second premolar, as shown by red arrows, and an unerupted third molar, as shown by black arrows.

**Figure 6:**
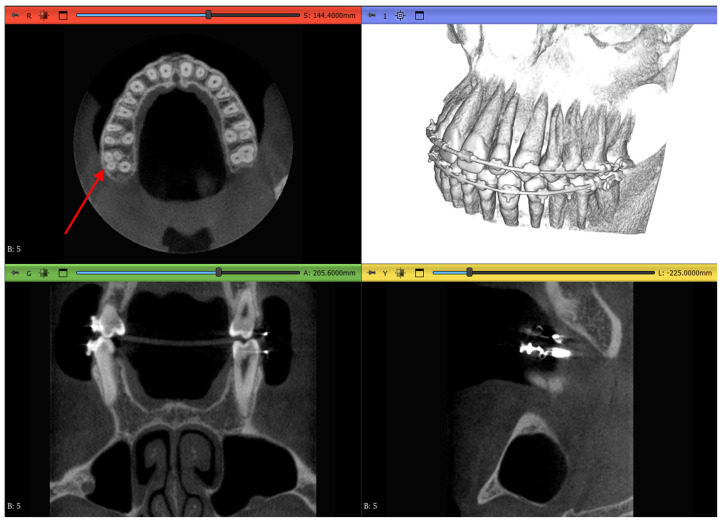
Dataset 3 of the study with a red arrow pointing to the 4-rooted maxillary second molar.

Dataset 3 presents the most challenging obstacle to the workflows, as it includes braces, and the maxillary second molar chosen has 4 roots instead of the more common 3-root configuration, as shown in Figure 6 by the red arrow.

These variations in the input datasets were chosen specifically to test various scenarios that could affect the applicability of the evaluated workflows, especially the automated and semi-automated ones.

### 2.2. Methodology

The proposed workflow aims to obtain near-perfect 3D models of maxillary central incisors and maxillary second molars from the three CBCT datasets using complete manual segmentation to ensure the most accurate representation, with these models used as the reference of comparison for all other extracted models by other workflows. The incisor is of a relatively regular shape, and, therefore, it is an easier test for non-manual methods. The molar is mainly chosen since it is most common for it to have three root canals [66]. Therefore, this should better demonstrate the strengths and weaknesses of each method, since multiple roots in teeth segmentation is sometimes the cause of inaccuracies in non-manual methods [34]. In addition, choosing two sets of teeth anatomies across three datasets is to verify that the proposed methods are not overfitted to just a single tooth or a single dataset. Other than the benchmark model for each tooth in each dataset, the following four other workflows are used to segment the same benchmark models, with six trials per workflow per tooth anatomy per dataset:

- Complete manual segmentation
- GFS algorithm
- WS algorithm
- Automated DentalSegmentator

GFS and WS are both semi-automated approaches whose implementation in 3D Slicer provides simple, easy-to-use interfaces for the user to carry out segmentation. The DentalSegmentator extensions developed by Dot et al. [25], and discussed earlier in Section 1.1, are also easy-to-use tools that facilitate the segmentation process. The main reason for the addition of this automated method is the ability to maintain a certain standard or validity for the proposed methods by comparing the results directly with state-of-the-art performance outcomes from recent studies. Table 1 shows the number of models created by each workflow, amounting to 150 3D models. The last manual trials of each tooth in each dataset are used as benchmarks, as it was noticed that the user was becoming more familiar with the dataset and software after each trial, and therefore, it was assumed that the last manual segmentation would be the most accurate one.

Comparisons of all models, including the manual segmentation trials, against the benchmark were performed using a dice similarity metric approach available in the “SlicerRT” extensions inside 3D Slicer [67]. Two main lines of comparison were carried out; the first comparison was a general one evaluating the overall tooth shape while accounting for the void in the pulp chamber and root canals. The second comparison was mainly focused on the accuracy of the root canals’ segmentation, since endodontics-oriented approaches are generally more challenging. This was achieved by removing the upper part of each tooth at the same level for each trial and using the subgingival part in the comparison using the same dice similarity approach; this created another 150 3D models of just the subgingival part of the teeth, bringing the total number of models in this study to 300. After the dice scores were calculated, Kruskal–Wallis tests were performed to determine whether there existed a difference in the medians between the non-manual methods and the manual method. Subsequently, Nemenyi tests were also carried out to identify which groups were significantly different from the manual segmentation method.

### 2.3. Pre-Processing

**Figure 7:**
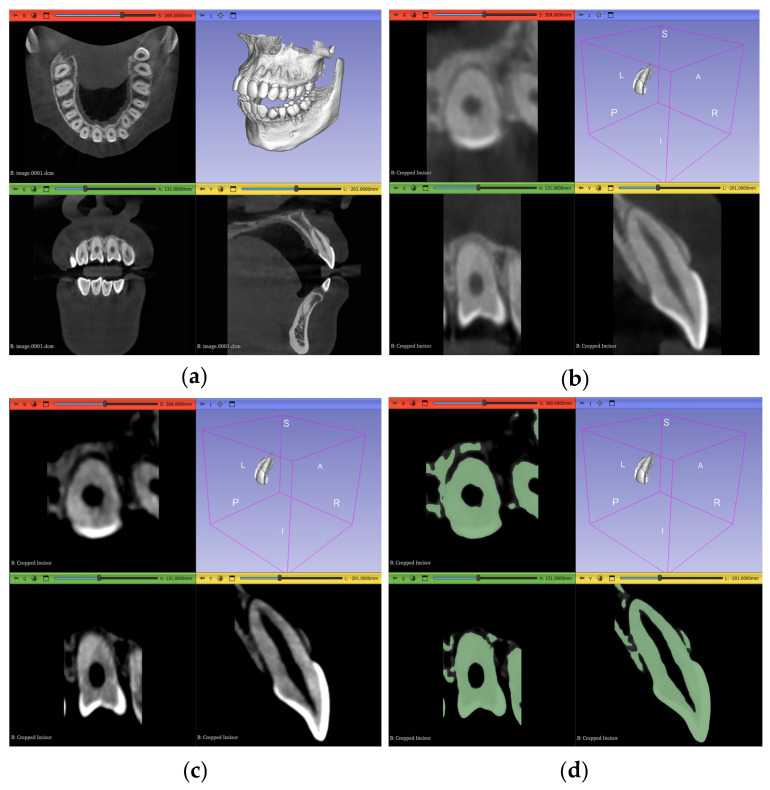
Pre-processing steps performed on the maxillary central incisor of Dataset 1: (**a**) dataset importing, (**b**) volume cropping, (**c**) histogram adjustment, and (**d**) applying a threshold mask.

**Figure 8:**
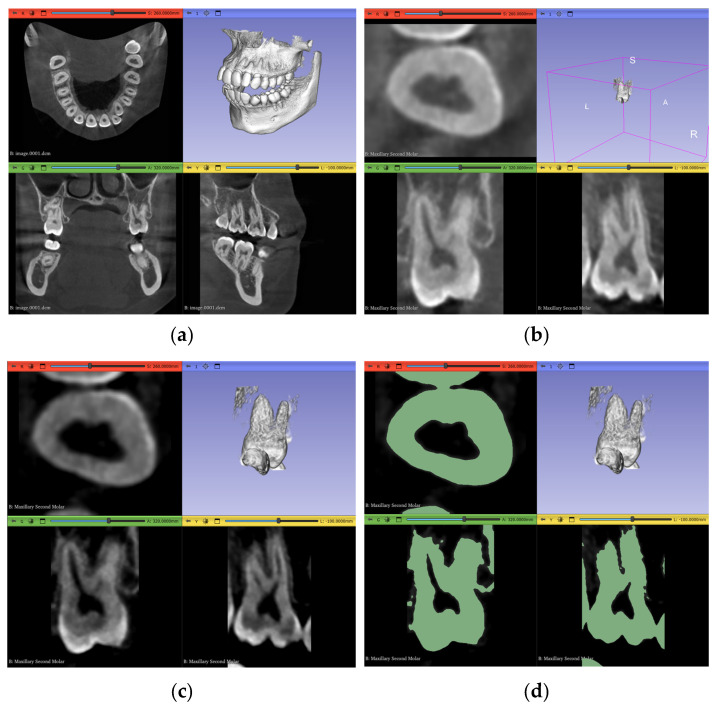
Pre-processing steps performed on the maxillary second molar in Dataset 1: (**a**) dataset importing, (**b**) volume cropping, (**c**) histogram adjustment, and (**d**) applying a threshold mask.

Regarding the actual segmentation procedures, some pre-processing steps were unified and applied to all trials, except the automated method, after importing the datasets, as shown in Figure 7a and Figure 8a, to ensure that any differences were solely due to the actual procedures proposed and not because of any external factors. The pre-processing steps included in the workflow were volume cropping, histogram adjustment, and threshold masking.

#### 2.3.1. Volume Cropping

After importing the dataset, it is convenient to crop the volume to only the area of interest. This is conducted using the “Crop Volume” module in the software. For a completely manual segmentation, cropping the volume is not of much significance; however, it might be quicker and more accurate for other computational approaches to deal with less data and, therefore, it becomes an important step. It is crucial to go through the entirety of the scan in the sagittal, coronal, and transverse planes after bounding the region of interest (ROI) to make sure the intended tooth does not exceed these boundaries and cause a loss of data in subsequent steps. The outcome of this step is shown in Figure 7b and Figure 8b.

#### 2.3.2. Histogram Adjustment

For better differentiation between darker and lighter pixels in the image, a histogram equalization process is one of the solutions. The “Volumes” module in the software is used in this step with the newly cropped volume as its input. Although it is an important step, a histogram that is too narrow will eliminate crucial information from the image. Therefore, the user also must go through the entire three planes during this step to guarantee no valuable pixels have been erased from the scan due to non-uniformity of the tooth density, either within itself or in reference to other teeth. Table 2 shows the values chosen in our specific datasets to adjust the histogram according to the subjective judgment that no valuable information is clipped outside the histogram range, while still offering a cleaner image with less noise than the original dataset. As demonstrated, the numbers will change even inside the same dataset for each ROI, depending on the desired outcome and judgment of the operator.

The results of the incisor and molar operations are shown in Figure 7c and Figure 8c, respectively.

#### 2.3.3. Thresholding Masking

This step is beneficial to limit the number of errors in consequent steps by using the thresholding tool in the “Segment Editor” module to create a mask of selectable pixels in the image based on the stated brightness level. This is substantially more useful for manual segmentation and iterations of seed correction. The threshold level itself should be selected based on the tooth of interest, as each tooth will have a different range of values. Two segmentation layers are created here, “tooth” and “non-tooth”, to which the mask is applied. The range selected for our datasets are shown in Table 3.

As shown in Table 3, the maximum value of the threshold mask is usually set to the maximum value of the available range, as the enamel part of the tooth most commonly represents the highest value of brightness due to its radiopaque nature. In Dataset 3, however, this is not the case; the brightest pixels are occupied by the metal braces and, therefore, the threshold range is clipped from the dark and bright sides of the scales The masks for the incisor and the molar are shown in Figure 7d and Figure 8d, respectively.

**Figure 9:**
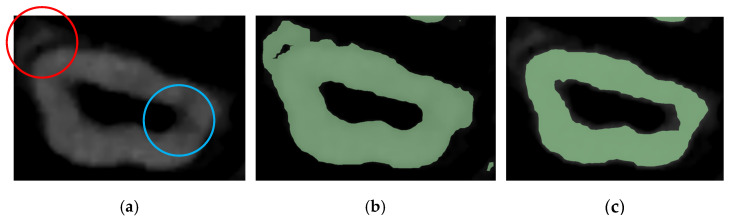
An example showing the judgment of preprocessing parameter selection: (**a**) molar before thresholding with red circle showing pixels to be discarded and blue circle showing pixels of interest, (**b**) conservative and inclusive parameter value of thresholding, and (**c**) aggressive and exclusive parameter value of thresholding.

All three pre-processing steps mentioned above will differ in parameter values depending on the subjectivity of the user and how inclusive the output of all necessary data should be. Figure 9a shows a slice of the maxillary second molar in Dataset 2 after the histogram adjustment, with 2 circled areas sharing similar brightness levels. The blue circle is an area of interest belonging to the tooth, while the red circle should not be segmented as part of the tooth. Figure 9b shows how an inclusive threshold minimum value should be selected by accepting the noise caused by the pixels in the red circle to gain selection access to the pixels in the blue circle, and Figure 9c shows a bad example of parameter selection for the threshold range, as part of the tooth is not covered despite the selection seeming cleaner and having less noise. The time taken to review that all operations are satisfactory and will not cause any data loss is around 10 min.

### 2.4. Workflow 1: Complete Manual Segmentation

3D Slicer offers manual pixel selection using either a paintbrush or a drawing technique, both of which are identical in terms of output results; however, one can be superior to the other depending on geometry. The painting brush tool has a circular selection area with an adjustable size, and the drawing tool lets the user line the perimeter of an area of interest to be included in the active segmentation layer. The painting process starts with choosing a plane and a slice at one of the ends of the desired tooth, which does not necessarily have to be the last slice if it is not clear because it will be more visible from another plane view.

**Figure 10:**
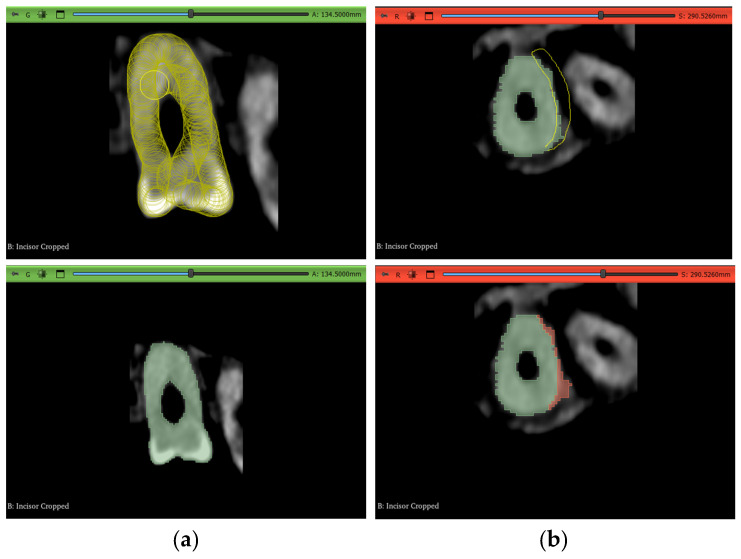
Manual selection of pixels using (**a**) circular paintbrush and (**b**) line drawing.

The tooth pixels were painted slice by slice in the first plane, and then a second view was used in the same manner, followed by a third view. After the initial segmentation stage was finished, alternating rounds of trimming and fine painting were performed from other views. It was noted that the brush tool was more time-efficient in large clusters of pixels with no interruptions; however, the drawing tool was more flexible for non-uniform slices, especially near the alveolar bone, where the threshold mask was not efficient as a barrier between pixels of teeth and bones. The brush tool is shown in Figure 10a, while the drawing tool in a ‘non-tooth’ segmentation layer is shown in Figure 10b. In Figure 10, yellow lines are the selection area, green pixels belong to the ‘tooth’ layer, and red pixels belong to the ‘non-tooth’ layer.

### 2.5. Workflow 2: GFS

**Figure 11:**
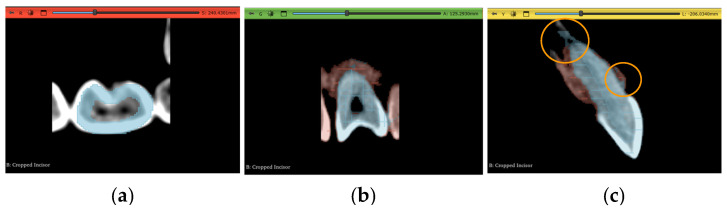
Summarizing takeaways of Experiment 2: (**a**) manual seeding of intermittent slices, (**b**) GFS algorithm initialization results, and (**c**) mislabeling performed by the algorithm.

This workflow is slightly similar to the work of [36]. Although their methodology is oriented toward periodontal applications, the same tool can be used with some modifications to account for root canal recognition and disregard of alveolar bone segmentation. In contrast to the manual method, this approach does not require manual painting in all slices, rather around ten slices per plane view, and it is also a fairly rough painting procedure that does not need to contain all the pixels in a slice. This technique is used to paint both layers, “tooth” and “non-tooth”, and is shown in Figure 11a.

Next is the initialization step of the GFS algorithm inside of 3D Slicer, which shows a preview of what the software thinks belongs to each segment of the input pixels in a slightly faded color, as shown in Figure 11b. Visual inspection is needed after that to assess whether the algorithm has correctly labeled the rest of the slices in between the input slices. If the labeling is not perfect, which is predictably the case, as shown inside the circles in Figure 11c, iterations of correction to the input slices are performed to better aid the algorithm in predicting which areas belong to which segment. In Figure 11, blue pixels belong to the “tooth” layer while red pixels belong to the “non-tooth” layer

**Figure 12:**
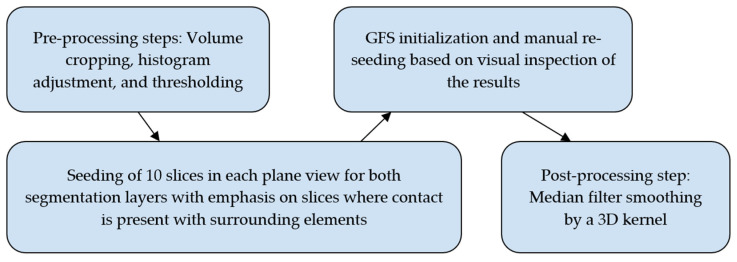
Overview of the steps taken in the GFS-based experiment for the incisor and molar teeth.

A balance has to be found between a perfect segmentation result and a satisfactory quick output. A summary of the steps is shown in Figure 12.

### 2.6. Workflow 3: WS

**Figure 13:**
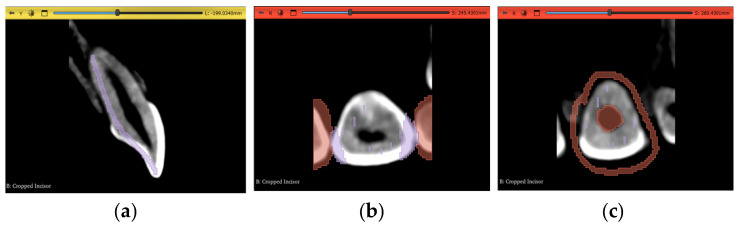
Summarizing takeaways of Experiment 3: (**a**) manual seeding of intermittent slices, (**b**) emphasis of seeds being on contact points by labeling the other segmentation layer, and (**c**) surrounding the tooth with the non-tooth segment.

In a similar fashion to the previous experiment, this workflow needs seeding in a few slices. The “tooth” segment was painted with narrow longitudinal strokes in the sagittal plane in a smaller number of slices than in Experiment 2, as shown in Figure 13a. Then, where points of contact between teeth were present, seeds were applied to both segments to differentiate between the two regions every three slices, as shown in Figure 13b, and the same strategy was applied in the subgingival part. It is important to note that the algorithm did not factor in the thresholding step explained in Section 2.3.3, and, therefore, it was required to entirely surround the tooth using the “non-tooth” segment. This is demonstrated in Figure 13c.

The Watershed tool, which is a part of the “SegmentEditorExtraEffects” extension, was used to then initialize the algorithm with the object scale value set to 0.2, which was selected based on the subjective judgment of the user so the results were efficient. Increasing the object scale value resulted in a smoother selection, since this value stands for a parameter important to edge detection and will inevitably lead to poor details in the segmented model. After applying the algorithm, the output was significantly cleaner than the grow-from-seed method. In Figure 13, the purple pixels belong to the “tooth” layer, and the red pixels belong to the “non-tooth” layer.

**Figure 14:**
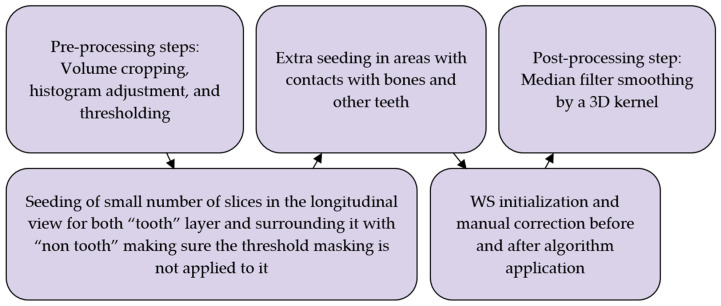
Overview of the steps taken in the WS-based experiment for the incisor and molar teeth.

Similar iterations to those in the previous experiment were used to enhance the updated algorithm selection; however, it did not take nearly as long. The steps of this experiment are shown in Figure 14.

### 2.7. Workflow 4: Automated DentalSegmentator

**Figure 15:**
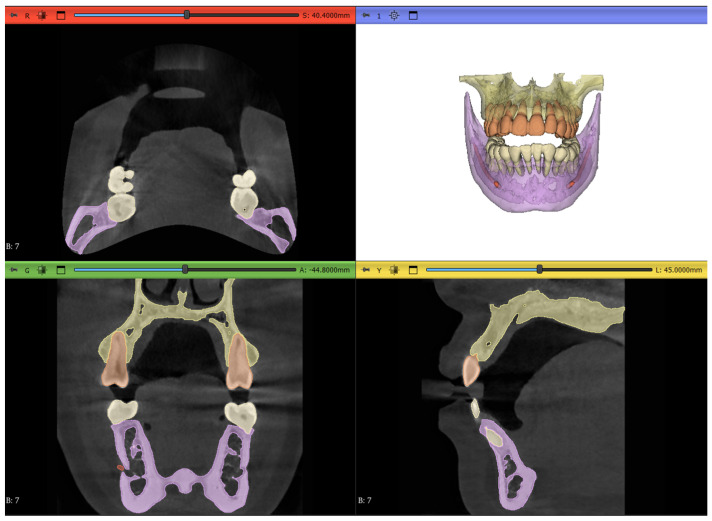
The output result of running the segmentation model on Dataset 1 of the study.

Developed by Dot et al. [25], this extension automatically segments maxillofacial CT or CBCT scans into the following 5 different segmentation layers, as shown in Figure 15:

- Maxilla and upper skull
- Mandible
- Upper teeth
- Lower teeth
- Mandibular canal

This extension offers an extremely simple interface with just one parameter to tweak in regard to surface smoothness; for our specific datasets, this parameter was kept at the default value of 0.5. There is also an option to run the model on the CPU if the computer does not have a cuda-compatible GPU; however, this will understandably take more time to complete.

**Figure 16:**
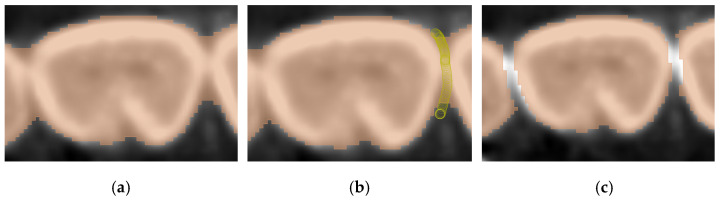
The results of segmentation using the automated model: (**a**) initial connection between adjacent teeth immediately after running the model, (**b**) erasing pixels of connection, and (**c**) isolated tooth of interest.

An acknowledged limitation of the extension by the authors is its inability to offer individual tooth labeling. Thus, a manual step was performed to isolate the tooth of interest by removing the connecting pixels to the adjacent teeth and then keeping the island of pixels belonging to the tooth while discarding all other islands. This is shown in Figure 16.

**Figure 17:**
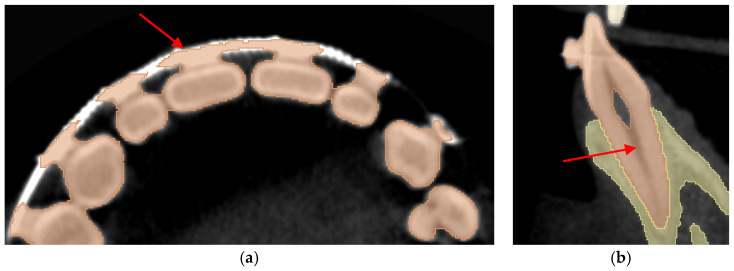
Limitations of the automated extension: (**a**) braces are considered part of the teeth and (**b**) root canals are not recognized, as shown by the red arrow.

Although some imperfections still persisted in the outputs of the segments, such as mislabeling of the orthodontic brackets and wires as part of the tooth or failing to recognize the root canals, as shown in Figure 17a and Figure 17b, respectively, no manual refinement was carried out to correct these errors. The reason for this decision was to fully test the autonomous nature of the extension and evaluate it against the manual segmentation. In Figure 17, the orange pixels belong to the “upper teeth” layer while the yellow pixels belong to the “Maxilla and upper skull” layer.

### 2.8. Post-Processing

**Figure 18:**
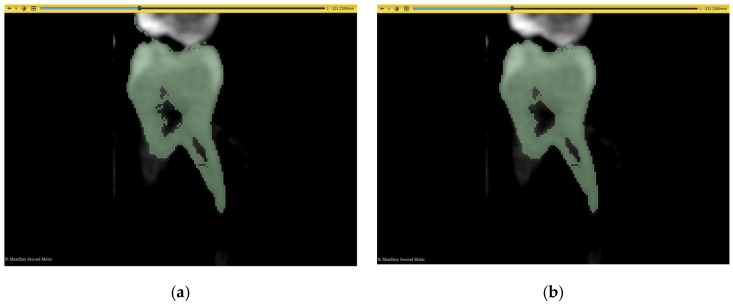
The effect of smoothing on a maxillary second molar: (**a**) imperfections visible on adjacent tooth and root canal before applying smoothing and (**b**) imperfections are removed after smoothing application.

After the acquisition of the 3D models, the resolution of each image, based on the scanning equipment, might introduce some sharpened edges and irregularities that deviate from the correct anatomy of the exported 3D model. Therefore, a polishing step must be introduced to all techniques, except the automated workflow, to ensure smooth surfaces in the model. 3D Slicer offers multiple ways to achieve this, one of which is the application of a 3D smoothing kernel. This is available to be performed using morphological operators such as opening and closing, a median approach, or a Gaussian approach. A median filter was used in this case with a kernel size of 3 × 3 × 3 pixels for all 3D models. It was noticed that the mm equivalent was not always consistent with the number of pixels due to the resolution of each input dataset, and that 3D Slicer also has a discrete stepping approach based on pixels and not mm. The rationale behind choosing this kernel size was that it is the minimum amount of smoothing to be applied mathematically, and, therefore, it did not risk removing any intricate details from the segmentation process. It is also worth noting that the smoothing effect was only applied to the “tooth” layer to remove imperfections such as those visible in Figure 18 in the adjacent tooth and root canal.

**Figure 19:**
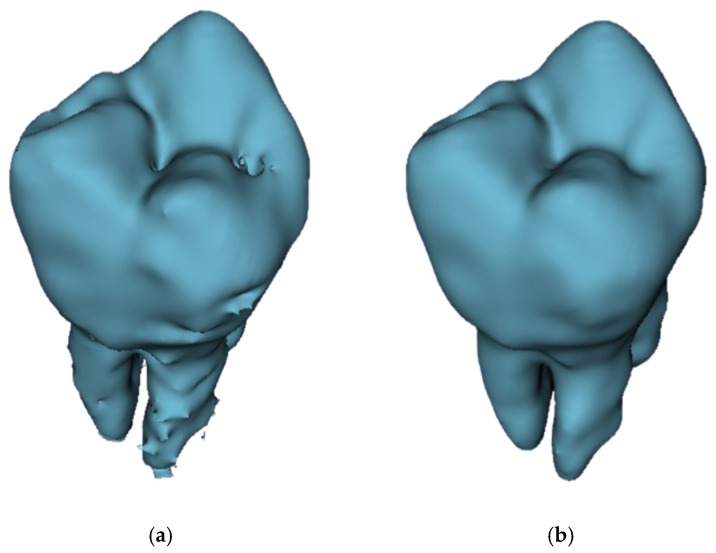
Three-dimensional visualization of smoothing effect on a GFS-obtained model: (**a**) before applying smoothing and (**b**) after applying smoothing.

Choosing not to apply the median filter to the “non-tooth” layer is due to the fact that the root canal section of this layer is often less than 3 pixels wide, and applying the kernel can remove the root canal completely and solidify the “tooth” layer. The results applied to a 3D model of a molar from Dataset 1 are shown in Figure 19.

**Figure 20:**
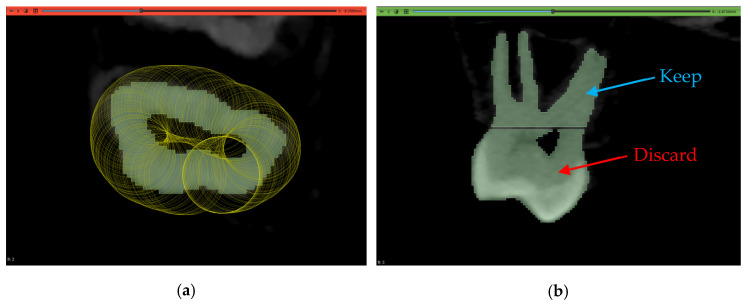
The method of splitting the model to obtain the subgingival part: (**a**) erasing on layer in the transverse plane and (**b**) the ouput of erasure to split the model in the sagittal plane.

After obtaining the 3D models, they were separated at the same height level using a similar isolation and island removal technique as that used in Section 2.5 to cleanly cut the models from the same plane. This is shown in Figure 20 for the maxillary second molar of Dataset 2. This process yielded another 150 models for the second line of comparison regarding the subgingival part of the teeth.

## 3. Results

**Figure 21:**
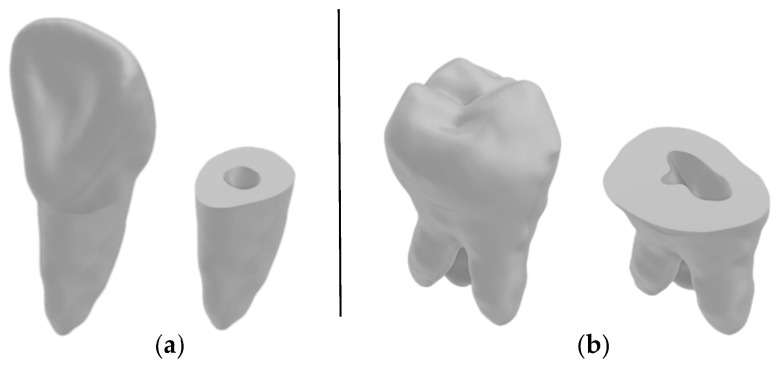
Final 3D reference models from Dataset 1: (**a**) the maxillary central incisor and (**b**) the maxillary second molar.

Figure 21 shows the final two 3D models from Dataset 1, with cross-sections showing successful segmentation with root canal recognition.

### 3.1. Three-Dimensional Model Comparisons

After all 3D models are obtained from 3D Slicer, the following comparisons are carried out and repeated for each dataset using the dice similarity coefficient:

- Incisor model from all four workflows against the benchmark incisor model,
- Incisor root model from all four workflows against the benchmark incisor root model,
- Molar model from all four workflows against the benchmark molar model,
- Molar root model from all four workflows against the benchmark molar root model.

### 3.2. Statistical Analysis

The dice similarity coefficient is a crucial metric that is widely used in the image segmentation community [68]. Therefore, a statistical difference test can use the dice scores in each workflow and compare them to the manual segmentation group, since manual segmentation is considered the “gold standard” of segmentation techniques [26,35,69]. Although ANOVA testing is great for this, it requires data to follow a normal distribution, which most of the groups do not, according to a Jarque–Bera test that was conducted. Thus, a Kruskal–Wallis test is chosen to find any statistically significant differences between the groups. A Nemenyi test is subsequently performed to determine which of the groups deviate from the manual group. All tests are performed using Microsoft Excel Version 2508 via “Real Statistics” plug-in [70].

### 3.3. Results of Dataset 1

**Figure 22:**
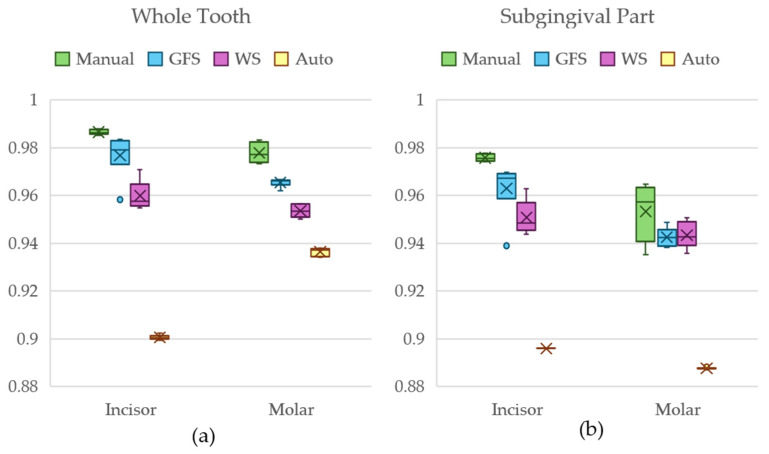
Box-and-whiskers plot of the dice score comparison result from Dataset 1 for (**a**) the whole tooth comparison and (**b**) the subgingival part comparison.

Figure 22 shows a box-and-whiskers plot of the dice scores for Dataset 1 using the entire tooth anatomies, as well as the root part of the teeth.

After carrying out the Kruskal–Wallis test for the incisor and molar models of Dataset 1, a *p*-value of <0.001 was obtained, leading us to assume that there was a statistically significant difference between the groups.

Table 4 shows a summary of the results from Nemenyi’s post hoc test for the whole tooth comparisons of both incisors and molars.

According to the number of trials and the 0.05 alpha level, the critical *q*-value is 3.633 from the Studentized Range *Q* table [71]. The *q*-stats for the groups show that only the GFS-obtained models do not statistically differ from manual segmentation method models in the comparison of Dataset 1 for the whole tooth for both incisors and molars.

The Kruskal–Wallis test for the subgingival parts of the incisor and molar for Dataset 1 also returned a *p*-value of <0.001. Table 5 shows the relations between each group with the manual segmentation method.

From the results from Table 5 and critical *q*-value of 3.633, the GFS models did not demonstrate significant differences from the manual group, the WS models only differed in the incisor root and not the molar root, and the automated method statistically differed in both anatomies.

### 3.4. Results of Dataset 2

**Figure 23:**
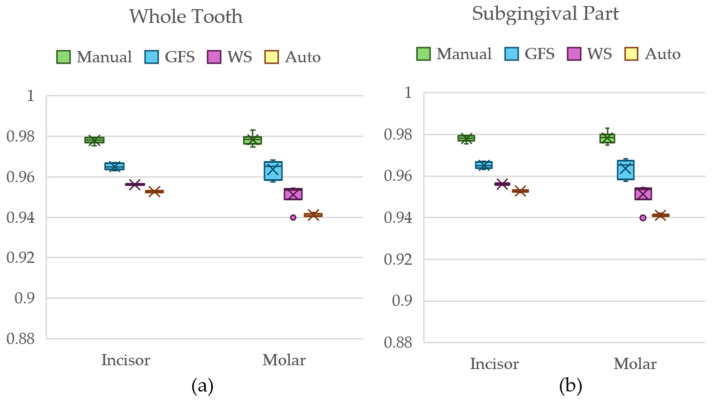
Box-and-whiskers plot of the dice score comparison result from Dataset 2 for (**a**) the whole tooth comparison and (**b**) the subgingival part comparison.

Figure 23 shows a box-and-whiskers plot of the dice scores for Dataset 2 using the entire tooth anatomies, as well as the root part of the teeth.

After carrying out the Kruskal–Wallis test for the incisor and molar models of Dataset 2, a *p*-value of <0.001 was obtained to conclude that a statistically significant difference between the groups existed. Table 6 shows a summary of the results from Nemenyi’s post hoc test for the whole tooth comparisons of both incisors and molars for Dataset 2.

With the critical *q*-value being 3.633, the *q*-stats from Table 6 show that only the GFS models did not statistically differ from those of the manual segmentation method for the whole tooth for both incisors and molars.

The Kruskal–Wallis test for the subgingival parts of the incisor and molar for Dataset 2 also returned a *p*-value of < 0.001. Table 7 shows the relations between each group with the manual segmentation method.

The post hoc results of the subgingival part and critical *q*-value of 3.633 allow us to conclude that WS-obtained models did not statistically differ from the manual group models for both anatomies, GFS only differed in the incisor anatomy model from manual models, and automated group models differed significantly in both anatomies.

### 3.5. Results of Dataset 3

**Figure 24:**
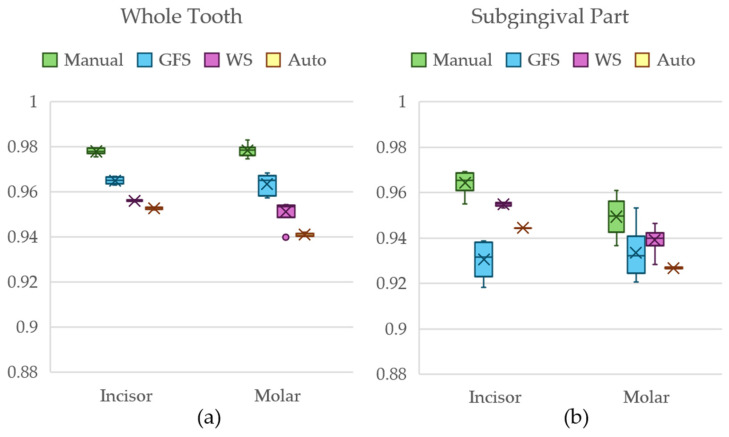
Box-and-whiskers plot of the dice score comparison result from Dataset 3 for (**a**) the whole tooth comparison and (**b**) the subgingival part comparison.

Figure 24 shows a box-and-whiskers plot of the dice scores for Dataset 3 using the entire tooth anatomies, as well as the root part of the teeth.

The Kruskal–Wallis test’s *p*-value of <0.001 for the incisor and molar models of Dataset 3 indicated the presence of a statistically significant difference between the groups. Table 8 shows a summary of the results from Nemenyi’s post hoc test for the whole tooth comparisons of both incisors and molars for Dataset 3.

Against the critical *q*-value of 3.633, the comparisons in Table 8 show that only the WS models did not statistically differ from those of the manual segmentation method for the whole tooth for both incisors and molars, GFS only differed in the incisor anatomy from the manual group, while the automated models differed in both anatomies from the manual group.

The Kruskal–Wallis test for the subgingival parts of the incisor and molar for Dataset 3 also returned a *p*-value of <0.001. Table 9 shows the relations between each group with the manual segmentation method.

According to the critical *q*-value of 3.633 and Nemenyi’s test results, it is observed that WS models did not statistically differ from those of the manual segmentation method for the subgingival part of both incisors and molars, GFS only differed in the incisor anatomy from the manual group, while the automated models differed in both anatomies from the manual group.

## 4. Discussion

Despite the statistical differences shown between the groups, all methods performed adequately in comparison to other reported dice scores from previous work [34]. Different applications of the segmented models might dictate stricter differences than others; for example, for a purely demonstrational application of the 3D model, such high similarity results are not typically required, while, for instance, an interactive VR simulator will definitely need accurate segmentation to be able to effectively train users. Another factor that should be discussed is the time taken to segment these teeth, which is often an approximation of the manual labor that went into the output. Table 10 shows the average numbers for each workflow per tooth anatomy across all datasets.

### 4.1. Manual Segmentation Method

The main benefit of manual segmentation is that it can produce a near-exact replica of an actual tooth. With enough iterations and time spent perfecting the boundaries created, every pixel of interest will be in the selection layer. Another advantage of this technique is that it is well suited for patient-specific segmentation, such as the tooth mentioned in Dataset 1 in Section 2.1, abnormally shaped, traumatized teeth, and CBCTs, including orthodontic devices such as Dataset 3, because it does not follow any fixed computation technique. Complete manual segmentation is powerful to use as ground truth training for CNN-based models, which makes it a relevant technique to be used despite the following disadvantages.

On the other hand, manual segmentation is evidently tedious and time-consuming, despite resulting in the most accurate outcome, as three planal sets of images need to be painted appropriately. A realistic scenario, however, is that by the end of the second plane, the third plane would have been almost painted and can be used as a verification step. The number of images, or slices, in each plane is determined by the equipment that was used in obtaining the CBCT and its settings. A common reference for slice thickness is around 0.2 mm [72]. An average maxillary central incisor has a mean width of 8.74 mm and a length of 9.84 mm, which produces 34 slices in one plane and 44 in another [73]. It becomes readily apparent that this method is inefficient to use as a main segmentation technique, especially when it comes to scalability and reproducibility. It also depends largely on the familiarity of the user with the software and their knowledge of the correct anatomy, which introduces subjectivity into the procedure.

### 4.2. GFS Method

The Kruskal–Wallis and post hoc testing failed to reject any statistical difference between the GFS method and the manual segmentation technique in any of the molar models, either in the whole tooth or in just the subgingival part. In regard to the incisor models, the tests showed a statistically significant difference between GFS and the manual groups in three out of six comparisons. This suggests that this technique could be used for some accuracy-dependent teeth segmentation applications, with caution in especially difficult or abnormal scans. Another advantage of this method is the reduced time compared to the manual segmentation method, with time savings as much as 47% and 56%, on average, for the incisor and the molar, respectively. It is also user-friendly, as the algorithm does not require any parameters to be set and, therefore, will output relatively similar predictions for the same density and location of input seeds. Another advantage is that it is computationally inexpensive, and it shows the updated preview automatically. It is also worth noting that the time spent using this method follows a decreasing trend as the user becomes more familiar with both the software and the dataset itself.

The noise produced after applying the GFS algorithm is its main disadvantage, as it forces the user to review quite a lot of the slices again to fix any mistakes committed by the algorithm. This significantly increases the segmentation time, as well as the weight of subjectivity, in what is supposed to be a semi-automated procedure. This is more dominantly observed in the molar trial.

### 4.3. WS Method

**Figure 25:**
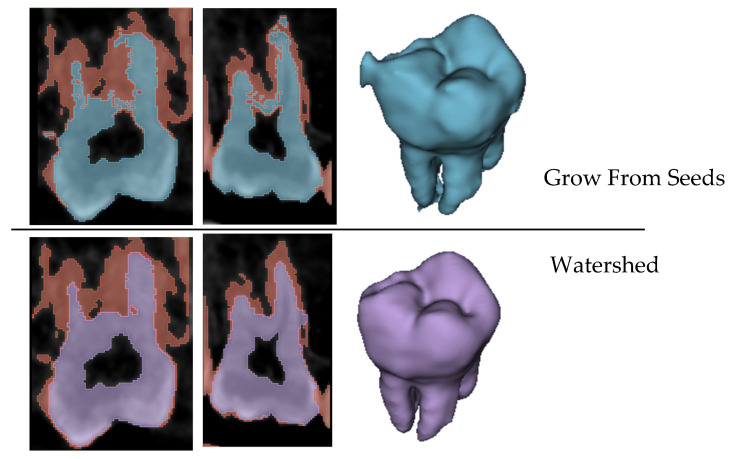
Two slices from the GFS and WS methods immediately after algorithm initialization and the output 3D model of each based on the input seeds.

The WS algorithm is well suited for segmenting irregular boundaries of objects due to its intrinsic nature of considering gradient information. It produces a significantly cleaner segmentation prediction after its initialization. This reduced the time spent during manual iteration. Statistical analysis showed no difference between the outputs of this method from the manual group in three of six incisor model comparisons and in four of six molar model comparisons. Time savings reached 58% and 60%, on average, for the incisor and the molar, respectively. Figure 25 shows the difference in prediction between GFS and WS immediately after initialization for the same two slices and in 3D. This helped minimize the subjectivity and time spent on correcting the mistakes committed by the algorithm. Another advantage is that the number of input seeds was much lower than the GFS approach outlined by Verykokou et al. [36], once again improving the duration of segmentation.

The main disadvantages of WS are the results of the Kruskal–Wallis and Nemenyi testing. Although it showed no statistical significance difference from the manual groups in some of the tests, it was slightly less consistent in that regard compared to the GFS method. Additionally, although it was quicker to edit the segmentation layers after initializing the algorithm due to a cleaner output, updating the input seeds was not as intuitive as the GFS method. The algorithm usually needed multiple consequent slices of manual reseeding to recognize mislabeling and correct it, unlike the GFS approach, which usually needed less manual refinement. Therefore, the WS method did not capitalize on the superior initialization stage. This dictated some manual refinement even after the algorithm had been applied, which was more time-efficient than updating the algorithm. Moreover, this method is dependent on the object scale parameter, which can vary the results significantly and, therefore, might introduce some delay while experimenting with this value.

### 4.4. Automated DentalSegmentator

As expected, the automated nature of this method makes it the easiest workflow by far. Not only that, but the model also takes the least time to complete the segmentation. The most significant advantage of this approach is the batching and scalability of output segments. In about 3–5 min, depending on the size and resolution of the input scan, the model yields five segments, as mentioned in Section 2.5, that extend to the hard tissue, as well as upper and lower teeth. Although there is evidence of statistically significant differences in this method’s models among comparisons of the manual group’s output, the overall shape, especially the crown part, is clean and of good details. The extension offers a single parameter which relates to the final surface smoothness and not the segmentation algorithm itself. This is mainly beneficial for beginners and is largely due to the fact that the underlying nnU-Net architecture is a self-configuring pipeline with regard to the required preprocessing steps and training parameters. This self-configuration nature allowed the model to successfully segment Dataset 2 with acceptable initial results, despite all the difficult anatomies mentioned in Section 2.1. This also meant that no independent preprocessing or postprocessing steps were performed, which contributed to a more streamlined workflow. The average time savings compared to the manual segmentation method were as much as 68% and 84% for the incisor and molar, respectively.

**Figure 26:**
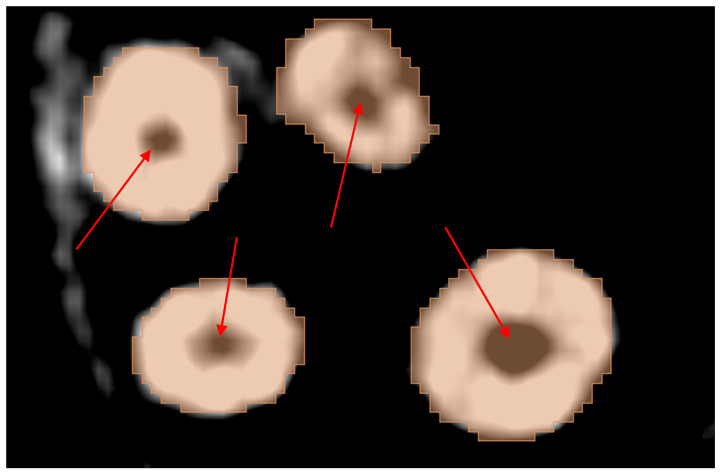
The limitation of the automated method in recognizing the root canals visualized by the red arrows.

Although visually, the results looked decent in most cases, the statistical testing showed statistical differences in all comparisons made from the manual segmentation groups. Moreover, upon closer inspection, root canal recognition was not of an acceptable level from the point of view of an endodontics-oriented application. Figure 26 shows the four roots in the molar of Dataset 3: mesiobuccal, distobuccal, mesiopalatal, and distopalatal roots. In this figure, the histogram is aggressively modified to better visualize the automated model’s mistake in mislabeling the hollow root canals as part of the solid dentin of the tooth. Having to manually correct this error would be a tedious process and would take away the main benefits of this method, which are autonomy and time savings; at this point, the extension becomes another semi-automated method and not a fully automated one as regarded. The model also failed to account for the orthodontic brackets and wire when segmenting the upper and lower teeth, which raises concerns about other patient-specific datasets and the model’s applicability for such cases.

As acknowledged by Dot et al. [25], “DentalSegmentator” currently does not offer individual teeth labeling, which can be problematic for some applications and would need further manual intervention, as demonstrated in this work in Section 2.5. The authors referenced other work that overcomes this issue; however, it does not overcome the issue of root canal recognition [7,74,75]. Lastly, the use of and reliance on such automated methods still do not conform to strict and optimal guidelines for safe application against automation bias and manual supervision [25].

## 5. Conclusions

The semi-automated dental image segmentation workflows introduced in this study, namely GFS and WS, demonstrate significant advantages in rapid and reliable ground truth acquisition for tooth and root canal segmentation, addressing one of the most challenging bottlenecks in training deep learning models for dental imaging. The results indicate that the GFS workflow achieves a segmentation accuracy nearly equivalent to manual methods for molars and only minor differences for incisors, while substantially reducing the labor and time required by almost half the effort compared to fully manual segmentation. The WS approach, though slightly less accurate, offers even greater speed and remains suitable for educational or anatomical applications where absolute precision is not critical. The DentalSegmentator automated model provides notable time savings, but its limitations in root canal identification and individual tooth labeling currently restricts its use for clinical and simulation training purposes, highlighting the continued importance of expert involvement in dataset curation. This work confirms that semi-automated segmentation enables faster dataset expansion for convolutional neural network development in dental applications, paving the way for broader adoption of AI-based analysis in dentistry while emphasizing the need to validate workflows against specific requirements, such as clinical planning or VR simulator realism. Future research should systematically evaluate the impact of ground truth dataset sources, whether semi-automated or fully manual, on model accuracy across diverse cases and further enhance automated frameworks to better identify complex root canal structures and accommodate dental anatomical variability and patient-specific cases. Standardized protocols for workflow validation and adaptation will increase their clinical and research utility, promoting the transferability and reliability of these promising segmentation approaches. In summary, validated semi-automated methods provide a scalable solution for high-quality dental dataset generation, strategically supporting the advancement of artificial intelligence in endodontics, clinical planning, and immersive educational technologies.

## Figures and Tables

**Table 1:** The number of 3D models segmented grouped by datasets, teeth anatomies, and workflows.

Datasets	Manual	GFS	WS	Auto
Dataset 1	7 Incisor (including 1 benchmark) 7 Molars (including 1 benchmark)	6 Incisors 6 Molars	6 Incisors 6 Molars	6 Incisors 6 Molars
Dataset 2	7 Incisor (including 1 benchmark) 7 Molars (including 1 benchmark)	6 Incisors 6 Molars	6 Incisors 6 Molars	6 Incisors 6 Molars
Dataset 3	7 Incisor (including 1 benchmark) 7 Molars (including 1 benchmark)	6 Incisors 6 Molars	6 Incisors 6 Molars	6 Incisors 6 Molars
Total Models	42 models	36 models	36 models	36 models
	150 models			

**Table 2:** The parameter values of the histogram adjustment grouped by tooth anatomy and dataset.

Dataset	Incisor		Molar	
	Window	Level	Window	Level
Dataset 1	1600	1400	3000	1600
Dataset 2	2300	1800	2100	1500
Dataset 3	4500	2800	3500	2400

**Table 3:** The parameter values of threshold masking grouped by tooth anatomy and dataset.

Dataset	Incisor		Molar	
	Min	Max	Min	Max
Dataset 1	1000	7820 (Max)	600	7827 (Max)
Dataset 2	750	3608 (Max)	600	2549 (Max)
Dataset 3	800	4200	9300	5300

**Table 4:** Summary of statistical analysis for the whole part of the tooth in Dataset 1.

Group 1	Group 2	Tooth Anatomy	*q*-Stat	Significantly Different
Manual	GFS	Incisor	2.194	No
		Molar	2.078	No
Manual	WS	Incisor	4.041	Yes
		Molar	4.157	Yes
Manual	Auto	Incisor	6.235	Yes
		Molar	6.235	Yes

**Table 5:** Summary of statistical analysis for the subgingival part of the tooth in dataset 1.

Group 1	Group 2	Tooth Anatomy	*q*-Stat	Significantly Different
Manual	GFS	Incisor	2.425	No
		Molar	1.612	No
Manual	WS	Incisor	3.81	Yes
		Molar	1.501	No
Manual	Auto	Incisor	6.235	Yes
		Molar	5.196	Yes

**Table 6:** Summary of statistical analysis for the whole part of the tooth in dataset 2.

Group 1	Group 2	Tooth Anatomy	*q*-Stat	Significantly Different
Manual	GFS	Incisor	2.078	No
		Molar	2.078	No
Manual	WS	Incisor	4.157	Yes
		Molar	4.503	Yes
Manual	Auto	Incisor	6.235	Yes
		Molar	5.889	Yes

**Table 7:** Summary of statistical analysis for the subgingival part of the tooth in Dataset 2.

Group 1	Group 2	Tooth Anatomy	*q*-Stat	Significantly Different
Manual	GFS	Incisor	5.889	Yes
		Molar	3.233	No
Manual	WS	Incisor	1.386	No
		Molar	3.349	No
Manual	Auto	Incisor	3.811	Yes
		Molar	5.889	Yes

**Table 8:** Summary of statistical analysis for the whole part of the tooth in Dataset 3.

Group 1	Group 2	Tooth Anatomy	*q*-Stat	Significantly Different
Manual	GFS	Incisor	3.811	Yes
		Molar	2.309	No
Manual	WS	Incisor	2.425	No
		Molar	3.406	No
Manual	Auto	Incisor	6.235	Yes
		Molar	6.062	Yes

**Table 9:** Summary of statistical analysis for the subgingival part of the tooth in Dataset 3.

Group 1	Group 2	Tooth Anatomy	*q*-Stat	Significantly Different
Manual	GFS	Incisor	6.004	Yes
		Molar	3.406	No
Manual	WS	Incisor	1.617	No
		Molar	1.617	No
Manual	Auto	Incisor	3.926	Yes
		Molar	4.907	Yes

**Table 10:** Time durations and corresponding averages for all trials, experiments, and teeth anatomies.

Group 1	Tooth Anatomy	Average Time (Min)
Manual	Incisor	19
	Molar	45
GFS	Incisor	10
	Molar	20
WS	Incisor	8
	Molar	18
Auto	Incisor	6
	Molar	7

## Data Availability

The raw data supporting the conclusions of this article will be made available by the authors on request.

## Abbreviations

The following abbreviations are used in this manuscript:

CBCT	Cone Beam Computed Tomography
GAN	Generative Adversarial Network
GFS	Grow From Seeds
WS	Watershed
DICOM	Digital Imaging and Communications in Medicine
ROI	Region of Interest
